# Revisiting Short Gastric Division for Laparoscopic Nissen Fundoplication: A Literature Review

**DOI:** 10.7759/cureus.96724

**Published:** 2025-11-12

**Authors:** Mostafa Mahran, Oday Al-Asadi, Almoutuz Aljaafreh, Rofida Sobh

**Affiliations:** 1 Upper Gastrointestinal Surgery, Homerton University Hospital, London, GBR; 2 General Surgery, Homerton, London, GBR; 3 Upper Gastrointestinal Surgery, King's College Hospital, London, GBR; 4 Family Medicine, Ain Shams University, Cairo, EGY

**Keywords:** anti-reflux surgery, dysphagia, fundal mobilization, gas‑bloat, laparoscopic, nissen fundoplication, reflux recurrence, short gastric vessels

## Abstract

The debate continues over whether short gastric vessels (SGVs) should be divided during laparoscopic Nissen fundoplication (LNF) to alleviate the wrap tension. This study aimed to narratively compile evidence from laparoscopic-only studies (2005-2025) that compared the effects of dividing versus preserving the SGVs on dysphagia, gas-bloat syndrome, and reflux recurrence. We synthesized randomized trials, meta-analyses, and guidelines published between 2005 and 2025 comparing SGV division versus preservation. Across long-term randomized follow-up and multiple meta-analyses, routine SGV division did not reduce dysphagia or improve reflux outcomes. It was associated with more gas-bloat and slightly longer operative time. In contemporary LNF, using a standardized technique, routine SGV division is unnecessary, and selective division is reserved for cases with clear tension.

## Introduction and background

Laparoscopic Nissen fundoplication (LNF) is a well-established procedure for adults suffering from chronic gastroesophageal reflux disease (GERD) that does not respond to optimized medical treatment. A common modification over the years has been the routine cutting of the short gastric vessels (SGVs) to enable a short, loose 360° wrap. However, as minimally invasive techniques have evolved to focus on complete hiatal repair, sufficient intra-abdominal esophageal length, and precise wrap construction, the necessity of routinely dividing SGVs has been called into question [1,2].

GERD presents a significant symptom and financial burden globally, with numerous patients still experiencing breakthrough regurgitation, coughing, or sleep disturbances even with optimized proton-pump inhibitor treatment. For patients who are appropriately selected and have objective evidence of pathological reflux, antireflux surgery can provide long-lasting symptom control and improve quality of life when executed with consistent technical precision [1-3].

The traditional reason for cutting the SGVs was to extend the reach of the fundus and reduce tension in the wrap, theoretically leading to a "floppy" fundoplication and lessening dysphagia. As surgical centers standardized essential procedures, such as complete hiatal dissection and repair, mediastinal esophageal mobilization for sufficient intra-abdominal length, and calibration using a large-bore bougie, many surgeons began to question the necessity of routinely dividing these vessels in most primary cases. Understanding the additional value (or lack thereof) of routinely dividing these vessels is crucial, as extra dissection can prolong surgery and increase bloating complaints without enhancing outcomes [1,2].

This review compiles current high-level evidence and translates it into practical, patient-focused recommendations for everyday surgical decision-making. Equally important is aligning surgical strategies with patient priorities, such as reducing dysphagia, minimizing bloating, and ensuring a quick return to normal activities, by concentrating on the most impactful factors. Therefore, this review emphasizes wrap calibration and personalized configuration, treating SGV division as an optional addition rather than a standard procedure [1,2].

## Review

Methods

Research databases, including MEDLINE (PubMed), Embase, and Cochrane CENTRAL, were searched from January 1, 2005, to October 21, 2025. The search strategy included a combination of controlled vocabulary and keywords related to “Nissen fundoplication”, “short gastric vessels”, “Rossetti”, “fundal mobilization”, “Toupet/partial fundoplication”, “dysphagia”, and “quality of life”. The criteria for inclusion were adult primary LNF with a specified SGV strategy or a Rossetti (no-division) comparison, English language or English abstract, full text availability, and outcomes such as dysphagia, gas-bloat, GERD-HRQL, pH-metry, reintervention, or durability. The study types given priority were randomized controlled trials, systematic reviews/meta-analyses, and adult guidelines (Table 1).

**Table 1:** Inclusion and exclusion criteria for study selection LNF: laparoscopic Nissen fundoplication, SGV: short gastric vessels, PPI: proton pump inhibitor, RCTs: randomized controlled trials

Inclusion criteria	Exclusion criteria
Adults undergoing LNF	Pediatric series
Comparison of SGV division vs preservation, or Nissen (division) vs Nissen-Rossetti (no-division), or assessment of non-division of SGV	Open surgery cohorts or mixed laparoscopic/open without separable data
Reported outcomes including dysphagia, gas-bloat, and/or reflux recurrence (symptoms, PPI use, or objective testing)	Non-English articles without an English abstract
Full-text available (English language or English abstract)	No accessible full text
Eligible designs: RCTs, cohort studies, systematic reviews/meta-analyses, and adult guidelines; conference abstracts only when methods and outcomes are clearly reported	Studies without relevant outcomes (no dysphagia, gas-bloat, or reflux recurrence data)

The initial search and hand-searching of references yielded 412 records. After deduplication and screening of titles/abstracts, 56 full texts were assessed. Twelve studies met the inclusion criteria for the qualitative synthesis. We synthesized the findings narratively and provided a summary table of the studies (Table 2). Two reviewers (M.M. and O.A.) independently screened the titles, abstracts, and full-text articles for eligibility. Disagreements between reviewers were resolved through discussion, and when consensus was not reached, a third reviewer (R.S.) was consulted to make the final decision. This process ensured objectivity and minimized selection bias in study inclusion.

**Table 2:** Included studies (2005-2025) Evidence levels per Oxford CEBM 2009: 1a: SR/meta‑analysis of RCTs, 1b: individual RCT, 2a: SR of observational studies/narrative SR, 2b: cohort, 5: expert opinion/technique LNF: laparoscopic Nissen fundoplication, SGV: short gastric vessels, QoL: quality of life, RCT: randomized controlled trial, ARS: antireflux surgery, GERD: gastroesophageal reflux disease, LES: lower esophageal sphincter, CEBM: Centre for Evidence‑Based Medicine

Study	Design/comparator	Key outcomes	Level of evidence (Oxford CEBM 2009)
Yang et al. 2008 [1]	Randomized controlled trial; SGV division versus no division during LNF	No difference in long-term dysphagia or reflux; higher bloating with division	1b
Kinsey-Trotman et al. 2018 [2]	Randomized controlled trial follow-up at ~20 years; SGV division versus no division in LNF	No advantage for division in reflux/dysphagia; more chronic bloating with division	1b
Kösek et al. 2009 [4]	Prospective randomized trial; SGV division versus no division in LNF	Equivalent reflux control; longer operative time with division	1b
Markar et al. 2011 [5]	Systematic review and meta-analysis of randomized trials; LNF with versus without SGV division	No clinical benefit for division; operative time increased with division	1a
de Mattos Farah et al. 2007 [6]	Randomized clinical trial; total fundoplication with fundal mobilization versus without SGV division	Short-term clinical outcomes similar; no functional benefit for routine division	1b
Engström et al. 2011 [7]	Combined analysis of randomized trials; division versus no division	Higher chronic gas-bloat with division; dysphagia and reflux similar	1a
Khatri et al. 2012 [8]	Meta-analysis (randomized/controlled studies); LNF with versus without SGV division	No functional gain with division; slight increases in length of stay and operative time	1a
Teixeira et al. 2015 [9]	Retrospective cohort; selective SGV division strategy during LNF	Division used in ~10%; outcomes comparable overall; supports selective approach	2b
Alemanno et al. 2017 [10]	Cohort study; long-term QoL after laparoscopic Nissen-Rossetti (no routine SGV division) anti-reflux surgery	Good long-term QoL; low burden of gas-bloat (context)	2b
Walle et al. 2019 [11]	Systematic review; persistent dysphagia after ARS	Dysphagia primarily related to wrap geometry rather than SGV handling	2a
Parsak et al. 2023 [12]	Comparative cohort; "floppy" Nissen (with fundal mobilization) versus Nissen-Rossetti (no routine SGV division)	Equivalent symptom control; division increased operative time	2b
Erol et al. 2024 [13]	Comparative cohort; LNF with SGV division versus Nissen-Rossetti (no division)	Quality of life similar; dysphagia slightly higher without division; reflux similar	

Results

Randomized trials and meta-analyses have consistently demonstrated no reduction in dysphagia or reflux recurrence with routine SGV division [4,5]. Division is associated with a higher prevalence of gas-bloat at medium- and long-term follow-up [6,7]. Selective division, employed in a minority of anatomically demanding cases, achieves a tension-free wrap without compromising outcomes [7].

Discussion

Across randomized trials, long-term follow-up, and meta-analyses, the early differences in dysphagia observed between strategies generally converge by 6-12 months as edema resolves and diet liberalization occurs. In contrast, the excess gas-bloat seen with routine division persists over time. These patterns suggest that wrap geometry, length tightness, and axial alignment, rather than fundal attachments, are the dominant determinants of venting and swallowing function [5,6]. Sensitivity analyses that exclude single-center series and earlier laparoscopic eras do not change the overall direction; effects remain neutral for reflux control and dysphagia, with a small but consistent increase in operative time when the SGVs are divided [1,7].

With the implementation of standardized procedures and bougie guidance, the incidence of dysphagia decreased in both the divided and preserved groups, highlighting that it is experience and protocolization, rather than the SGV policy, that influences outcomes [14]. Bougie size and wrap calibration are important confounding variables that may explain discrepancies in postoperative dysphagia reported across earlier studies. Early laparoscopic trials often used small-caliber bougies (<40 Fr), producing tighter wraps and higher dysphagia rates [1,4]. As techniques matured, calibration with a 50-60 Fr bougie became standard, ensuring a tension-free, “floppy” wrap and markedly reducing dysphagia in both divided and preserved groups [2,14]. This procedural standardization likely accounts for the loss of any perceived benefit from routine short-gastric-vessel division in modern series. Consequently, the primary determinant of postoperative swallowing function appears to be the accuracy of wrap calibration rather than SGV management. Current randomized and long-term studies conducted under these standardized conditions confirm equivalent outcomes between division and preservation strategies [2,7,14].

Gas-bloat syndrome refers to the disruption of venting at the gastroesophageal junction. Several RCTs and meta-analyses have demonstrated that routine division of the SGV is associated with an increase in chronic bloating and flatulence, without any improvement in reflux, thereby supporting the standard practice of preservation [1,6,7]. Patient-reported outcomes indicate that the ability to belch and vomit is better maintained when wraps are genuinely floppy; routine division does not improve venting and is linked to a greater bloating burden over time [4,5]. Initial treatment involves educating patients on behavioral techniques, such as eating more slowly, reducing air swallowing, and dietary changes. The surgical approach focuses on adjusting the wrap rather than dividing the SGV [15].

Objective assessments are consistent with clinical impartiality; postoperative pH-metry and LES pressure profiles show similar results between division and preservation, and minor numerical variations have not resulted in lasting patient-centered advantages [1,4]. Research employing standardized pH protocols reveals that the percentage of time with pH<4 and DeMeester scores are similar, regardless of the SGV strategy used. Additionally, manometry indicated comparable postoperative LES pressures and intra-bolus pressures, supporting the notion that routine division offers no mechanistic benefit [8].

Subgroup considerations

In reoperative areas, careful dissection around the splenic hilum is crucial. While selectively dividing structures can help orient the fundus behind the esophagus, this must be balanced against the risk of bleeding and the potential for a partial wrap to more effectively manage reflux and swallowing functions [2,9].

Selective division of the SGV is justified when there is persistent objective intraoperative tension following complete mobilization of the mediastinum and repair of the hiatus, particularly in cases of large paraesophageal hernias or when the fundus is anatomically large. Observational and comparative studies have advocated for a customized approach rather than a universal rule [13,16,17].

In patients with high BMI, routine division slightly extends the duration of surgery and may subject the short gastric branches and splenic capsule to traction-related injuries. Although modern energy devices enhance efficiency, their lack of clinical advantages suggests that additional dissection in primary cases is unnecessary [7]. Increased intra-abdominal pressure due to obesity complicates the construction of wraps, but there is no evidence to support routine division, specifically for obese individuals. The results depend on the quality of hiatal repair and calibration of the wrap [2,9].

Safety and perioperative metrics

Although bleeding and capsular injuries are infrequent, they are more likely to occur with additional dissection around the splenic hilum. Teams that have stopped performing routine division avoid this risk without compromising reflux control or patient satisfaction [1,6,7].

Division slightly extends the time needed for surgery and introduces a minor risk of bleeding at the splenic hilum; however, overall complications are minimal in contemporary studies and are not reduced by routine division [1,7].

Most initial symptoms can be alleviated through a structured approach that includes transitioning to a liquid or soft diet, avoiding carbonated drinks, and providing early reassurance. If dysphagia continues, a timed barium swallow can provide context for endoscopic results and inform the need for dilation; persistent mechanical issues should be addressed with specific revisions rather than attributing them to undivided SGVs [15,18].

Future directions

Future research should categorize patients by hernia size, esophageal length, and motility to identify micro-subgroups that could benefit from division. Comparative effectiveness studies should incorporate the cost and recovery metrics. In practice, this involves testing the position of the fundus after cruroplasty with the bougie in place, ensuring that the wrap is positioned without axial traction, and allowing a gentle passage of instruments. If reach is restricted, a staged selective division of the uppermost SGVs can be performed until sufficient laxity is achieved. If motility is marginal, a posterior partial wrap is preferred over further dissection.

## Conclusions

Routine division of the SGVs during LNF conferred no advantage in dysphagia or reflux outcomes and increased complaints of gas bloating. Default preservation with selective division only, to relieve clear intraoperative tension, aligns best with high-level evidence and current guidelines.
